# Asthma and COPD Overlap Syndrome (ACOS): A Systematic Review and Meta Analysis

**DOI:** 10.1371/journal.pone.0136065

**Published:** 2015-09-03

**Authors:** A. Alshabanat, Z. Zafari, O. Albanyan, M. Dairi, J. M. FitzGerald

**Affiliations:** 1 Department of Experimental Medicine, The University of British Columbia, Vancouver, British Columbia, Canada; 2 Centre for Clinical Epidemiology and Evaluation, Vancouver Coastal Health Institute, Vancouver, British Columbia, Canada; 3 Department of Medicine, King Fahad Specialist Hospital, Dammam, Saudi Arabia; 4 Department of Medicine, The University of British Columbia, Vancouver, British Columbia, Canada; 5 Institute for Heart and Lung Health (IHLH), Faculty of Medicine, University of British Columbia, Vancouver, British Columbia, Canada; University of Dundee, UNITED KINGDOM

## Abstract

**Background:**

The combination of asthma and chronic obstructive pulmonary disease (COPD), or ACOS is a recently defined syndrome. The epidemiology of the condition is poorly described and previous research has suggested ACOS is associated with worse outcomes than either condition alone. We therefore decided to complete a systematic review of the published literature.

**Methods:**

This review was conducted in accordance with the Preferred Reporting Items for Systematic Reviews and Meta- Analyses guidelines. A structured search was performed in the PubMed, Embase, and Medline databases up to Feb 2015 to identify studies reporting incidence, prevalence, health care utilization, morbidity, or mortality in COPD and asthma.

**Results:**

A total of 19 studies were included in the present study. The pooled prevalence of overlap among COPD was 27% (95% CI: 0.16–0.38, p<0.0001) and 28% (95% CI: 0.09–0.47, p = 0.0032) in the population and hospital-based studies, respectively. We found no significant difference between ACOS and COPD in terms of gender, smoking status, lung function and 6mWD. However, in comparison to subject with only COPD, ACOS subjects were significantly younger, had higher BMI, healthcare utilization, and lower HRQoL.

**Conclusion:**

ACOS is a common condition that exists in a substantial proportion of subjects with COPD. ACOS represents a distinct clinical phenotype with more frequent exacerbations, hospitalization, worse health-related quality of life, and higher healthcare costs than either disease alone. There is a critical need to better define the management and treatment of this syndrome.

## Introduction

Asthma and Chronic Obstructive Pulmonary Disease (COPD) are major public health problems. Asthma and COPD are typically characterized as different diseases with unique epidemiological features as well as pathophysiological mechanisms. Asthma is an allergic disease that often develops during childhood, although it can also be diagnosed in adult life. It is characterized by airway hyper-responsiveness (AHR) that leads to intermittent and usually reversible airway obstruction, whereas COPD is a chronic respiratory disease that is typically linked to smoking tobacco, usually presents in subjects older than forty years of age, and is characterized by progressive and irreversible airway obstruction[1,2]. These definitions allow asthma and COPD to be recognized as distinct disease entities. However, this concept needs to be re-evaluated as many epidemiological studies have shown that asthma and COPD may coexist, or at least one condition may evolve into the other creating a condition commonly described as Asthma and COPD Overlap Syndrome (ACOS). It is a syndrome in which older adults with a significant smoking history have features of asthma in addition to their COPD[3]. However, the exact definition of this syndrome remains ambiguous as it characterized by a functional and pathological overlap between asthma and COPD[4]. In a recent document developed by the science committees of both the Global Initiative for Asthma (GINA) and the Global Initiative for Chronic Obstructive Lung Disease (GOLD), ACOS was defined as a syndrome characterized by persistent airflow limitation with several features usually associated with asthma and several features usually associated with COPD [5]. This overlap makes the differentiation between COPD and asthma with persistent airflow limitation difficult, especially in smokers and elderly people[3,6,7]. The objectives of this systematic review were to evaluate the prevalence of ACOS in COPD patients, to explore its main characteristics, and to determine the association between ACOS and the outcomes: exacerbation, hospitalization, health care utilization, and health related quality of life.

## Methods

### Search Strategy

This systematic review was conducted according to the guidelines of the Preferred Reporting Items for Systematic Reviews and Meta-Analyses (PRISMA) Statement[8] (see S1 PRISMA Checklist). A structured and comprehensive search of medical literature indexed in the electronic PubMed, Embase, and Medline databases was conducted using a detailed search strategy with a combination of free-text search terms and medical subject headings. The search strategy was discussed and agreed upon by two authors (Alshabanat A and JM. FitzGerald). The keywords search was conducted in April 2014 and updated in Feb 2015. Details of the search process are presented in S1 Fig.

### Review of studies

To determine study eligibility, the titles and abstracts of all search results were screened in two stages by two reviewers (Alshabanat A and Albanyan O) using predefined inclusion and exclusion criteria. Stage 1 involved reviewing all titles and abstracts to determine whether to include or exclude them, and Stage 2 involved reviewing the full text of the articles identified in Stage 1 to determine their eligibility for data extraction. Additional studies were identified by searching reference lists from primary studies and review articles.

#### Inclusion Criteria

Inclusion criteria included all published studies reporting incidence, prevalence, health care utilization, morbidity, or mortality in COPD and asthma.

#### Exclusion Criteria

Articles were excluded if they met at least one of the following exclusion criteria:

· excluded patients with COPD and/or asthma;
· did not report data on incidence, prevalence, health care utilization, morbidity, or mortality on asthma and COPD;
· were conducted in a specific sub-population with diseases that increased risk of respiratory disorders;
· were a commentary, an editorial, a case report, a letter. there were no published clinical trials in this population and by default this type of study was also excluded;
· was a duplicate of an article that had been previously identified.

### Quality Assessment

Two independent reviewers (Alshabanat A and Dairi M) evaluated the quality of the studies using the Newcastle-Ottawa scale (NOS)[9]. Studies could be awarded a maximum score of 9 points. Studies with scores of 5 points or more were considered to be of moderate to good study quality. However, all studies were used for analysis, irrespective of NOS score. Any disagreements were resolved by consensus.

### Data Extraction

Data-extraction was performed independently by two authors (Alshabanat A and Albanyan O) using a pre standardized data extraction form. For each accepted study[10–26], the following data, when available, were extracted: author, year of publication, country, study design, study setting, objectives, sample size, inclusion and exclusion criteria for participants, age range and mean age of study subjects, gender, smoking status (active, ex, and never smokers), diagnostic criteria, pulmonary function tests, use of medications, health care utilization, and number of cases (COPD alone, and overlap). Specific attention was given to the definition of the diagnoses of COPD and asthma. Any discrepancies were resolved by iteration and consensus. If essential data were lacking in the original studies, their authors were contacted.

#### Definition of the outcomes

Since ACOS does not yet have well-defined diagnostic criteria, we have adopted the definitions used in several epidemiological studies and reviews.[5, 7,14,19] We defined the overlap phenotype as any COPD patient with at least one or more of the following findings:

- Physician diagnosed asthma or self reported physician diagnosis of asthma
- Reversibility testing (≥ 12% and at least 200ml change in FEV1 from baseline).
- Peak Expiratory Flow (PEF) variability (≥ 20% change in PEF).
- Airway hyper-responsiveness to methacholine or histamine.

However, to avoid overestimation, we deliberately restricted the meta analysis to studies where COPD has been defined based on post-bronchodilator (BD) spirometric criteria.

### Statistical analysis

We pooled the prevalence of overlap among COPD patients using random effects models in the statistical platform R 3.1.0 [REF]. Forest plot was used to represent the details of each study. In addition, in separate meta-analyses we pooled the difference of the prevalence of overlap in males versus females.

## Results

**Fig 1:**
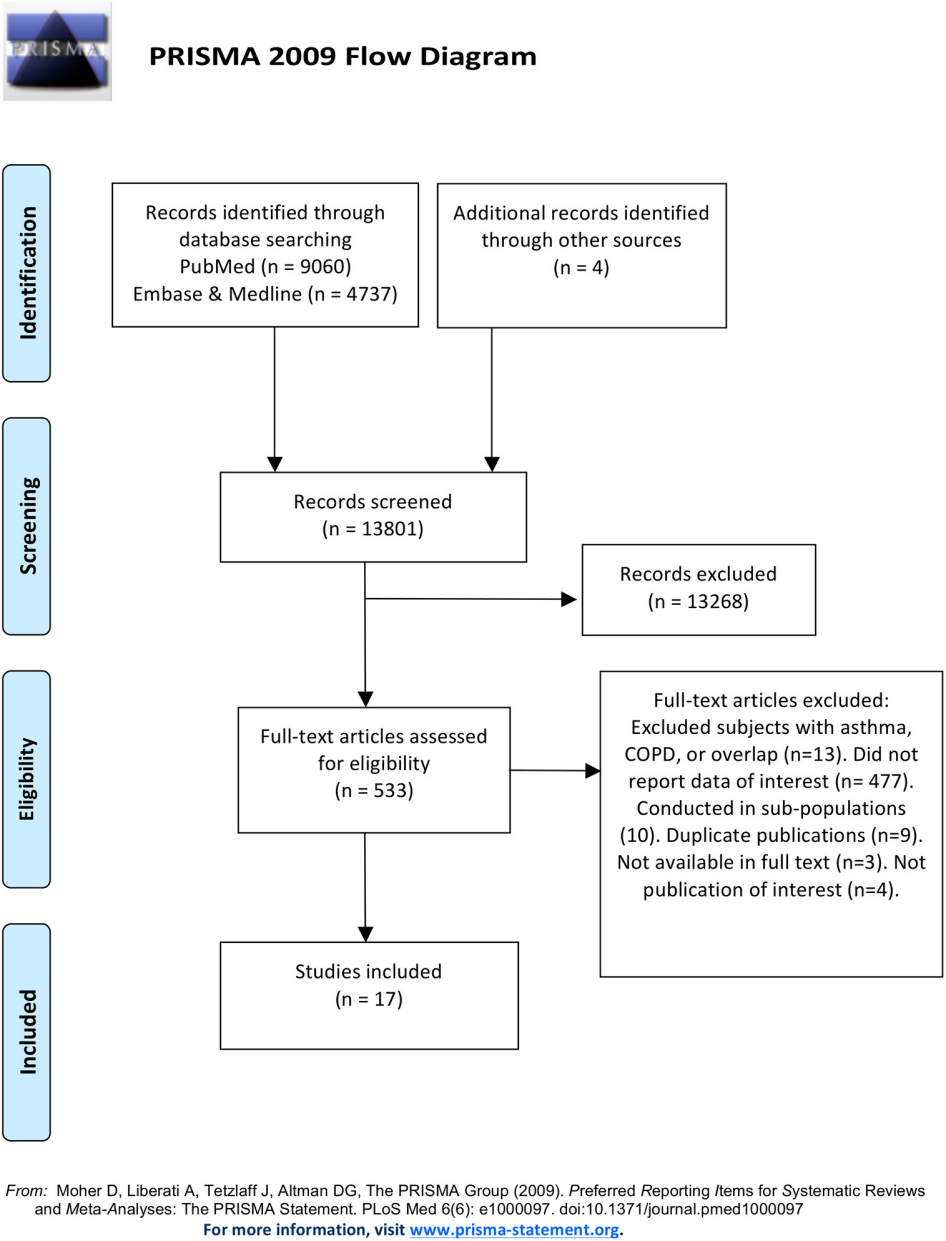
Review process (PRISMA Flow Diagram): details of review process.

The search resulted in 8,962 publications derived from PubMed, 4,737 publications from Embase and Medline, and 4 studies from reference lists. After title and abstract review, 13,170 studies were excluded. Of 533 studies meeting the initial inclusion criteria, 17 were accepted for data extraction. A detailed diagram of the review process is presented in Fig 1.

### Study Characteristics

Of the 17 included studies, 12 were cross sectional, and 5 were cohort studies. Based on study setting, 10 studies were population based, and the other seven were hospital based. The characteristics of the selected studies are summarized in Table 1.

**Table 1:** Characteristics of included studies.

Study	Study Center	Sample Size	Age	Diagnosis Method	Diagnosis Method	Result	Result	NOS
				Asthma	COPD	COPD only	ACOS	
**Shirtcliffe et all (2010) [10]**	New Zealand	749	25–75	1. Reversibility test (+ve if ≥ 12%/200ml change in FEV1), **Or** 2. Peak Expiratory Flow (PEF) variability (+ve if ≥ 20% change in FEV1), **Or** 3. Physician Diagnosed Asthma +Current symptoms, **Or** 4. Physician Diagnosed Asthma + inhaler use in the preceding 12 months	Post bronchodilator spirometry (FEV1/FVC <0·70)	51	65	7
**Menezes et al (2013) [11]**	Brazil, Chile, Mexico, Uruguay, Venezuela (PLATINO)	5,044	≥40	1. Wheezing in the last 12 months _+_ post-BD increase in FEV1 or FVC of 200 ml and 12%, **Or** 2. Self-reported prior diagnosis of asthma.	1. Post bronchodilator spirometry (FEV1/FVC <0·70), **OR** 2. Lower limit of normality (defined as the lower 5th percentile for predicted post-BD FEV1/FEV and FEV1/FVC).	594	89	6
**Marsh et al (2008)[12]**	New Zealand	469	65.4 (6.3)	1. FEV1 bronchodilator reversibility ≥15%, **Or** 2. Had peak flow variability ≥20%, **Or** 3. Had a physician’s diagnosis of asthma and (current symptoms and/or inhaler use).	Post bronchodilator spirometry (FEV1/FVC <0·70)	43	53	5
**Kauppi et al (2011) [13]**	Finland	1,546	56(12)	Based on the British Guidelines on Asthma Management with one or more of the following findings: a post bronchodilator increase in FEV1 of ≥12%, a bronchodilator response of ≥15% or diurnal variation of ≥20% in PEF recording, moderate to severe bronchial hyper-reactivity, or a decrease in FEV1 of ≥15% in the exercise test.	Post bronchodilator spirometry (FEV1/FVC < 0.70 OR FEV1/FVC < 88% of predicted reference value)	237	225	7
**Hardin et al (2011)[14]**	USA	915	45–80	Self Reported Physician Diagnosis of Asthma prior to the age of 40	Post bronchodilator spirometry (FEV1/FVC < 0.7 and FEV1 < 80% predicted)	796	119	5
**Alonso et al (2013) [15]**	Spain	331	> = 40	Physician Diagnosed Asthma before age 40	Post bronchodilator spirometry (FEV1/FVC <0·70)	291	40	5
**Johannessen et al (2005) [16]**	Norway	908	41 (15)	Self Reported Physician Diagnosis of Asthma	Post bronchodilator spirometry (FEV1/FVC <0·70)	30	10	6
**Danielsson et al (2010) [17]**	Sweden	548	≥40	Physician Diagnosed Asthma	Post bronchodilator spirometry (FEV1/FVC <0·70)	70	16	7
**Methvin et al (2008) [18]**	USA	508	≥40	Self Reported Physician Diagnosis of Asthma	Post bronchodilator spirometry (FEV1/FVC <0·70)	72	28	5
**Miravitlles et al (2013) [19]**	Spain	3,885	40–80	Self Reported Physician Diagnosis of Asthma	Post bronchodilator spirometry (FEV1/FVC <0·70)	318	67	5
**Zhou et al (2009)[20]**	China (CESCOPD)	20,245	≥40	Physician Diagnosed Asthma	Post bronchodilator spirometry (FEV1/FVC <0·70)	1477	191	5
**Kotaniem et al (2005) [21]**	Finland	683	21–70	Based on the following criteria: (self-reported asthma or had a positive skin-prick test) **and** (had reported attacks of respiratory obstruction or wheezing).	Post bronchodilator spirometry (FEV1/FVC <0·70)	52	12	6
**Fabrri et al (2002) [22]**	Italy	46	≥ 30	Physician Diagnosed Asthma	Post bronchodilator spirometry (FEV1/FVC <0·70)	27	19	6
**Rhee et al (2013)[23]**	Korea	185,147	> 40	1. ICD-10 codes for asthma (J45.x- J46), 2. Use of more than one drug for asthma at least twice per year (LAMA, LABA, ICS, ICS+LABA, SAMA, SABA, theophylline, LTRA, systemic corticosteroids, or systemic beta agonist).	1. Age greater than 40 years, 2) ICD-10 codes for COPD or emphysema (J42.x–J44.x, except J430), 3. Use of more than one drug for COPD at least twice per year (LAMA, LABA, ICS, LABA+ICS, SAMA, SABA, or Theophylline).	84143	101004	6
**Blanchette et al (2008)[24]**	USA	8,086	≥ 40	1. Age greater than 40 years, **and** 2. Have at least 1 medical claim with an ICD-9-CM code for COPD and at least 1 medical claim for asthma (493.0x, 493.1x, 493.8x, 493.9x) during the identification period **and** 3. Either (at least 1 additional claim with an ICD-9-CM code for COPD and asthma (493.2x) OR at least 1 additional medical claim for COPD and at least 1 additional claim for asthma within the observation period).	1.age greater than 40 years, **AND** 2. ICD-9 codes for COPD (491.xx for chronic bronchitis, 492.xx for emphysema, or 496.xx for chronic airway obstruction not classified elsewhere), **AND** 3. Have at least 1 additional medical claim with an ICD-9-CM code for COPD during the 24-month observation period.	6243	1843	6
**De marco et al (2010) [25]**	Italy	8360	20–84	The GEIRD Screening Questionnaire(Physician Diagnosed)	The GEIRD Screening Questionnaire (Physician Diagnosed)	431	175	5
**Shaya et al (2008)[26]**	USA	9131	≥ 40	1. Age 40 to 64 years, AND 2. At least 1 claim with ICD-9 code for Asthma (493.xx) in the primary, secondary, or tertiary diagnosis field.	1. Age 40 to 64 years, AND 2. At least 1 claim with ICD-9 codes for COPD (491.xx, 492.xx, 496.xx).	3455	2604	6

Abbreviations and definitions: COPD = chronic obstructive pulmonary disease; FEV1 = forced expiratory volume in 1 second; FVC = forced vital capacity, ICD: International Classification of Diseases.

### Quality Assessment

All included studies were assessed using the adjusted NOS scale (see S2 and S3 Figs for the adjusted NOS scales). Scores for the included studies ranged from 5 to 7, with a median of 6 points. Most studies matched or adjusted for some potential confounders, including age, gender, and smoking status. The quality assessment scores of selected studies are listed in S1 Table.

### Prevalence

Few studies have attempted to quantify the prevalence of ACOS, and prevalence rates between 15 and 55% have been reported among COPD patients. [3,12–14].

**Fig 2:**
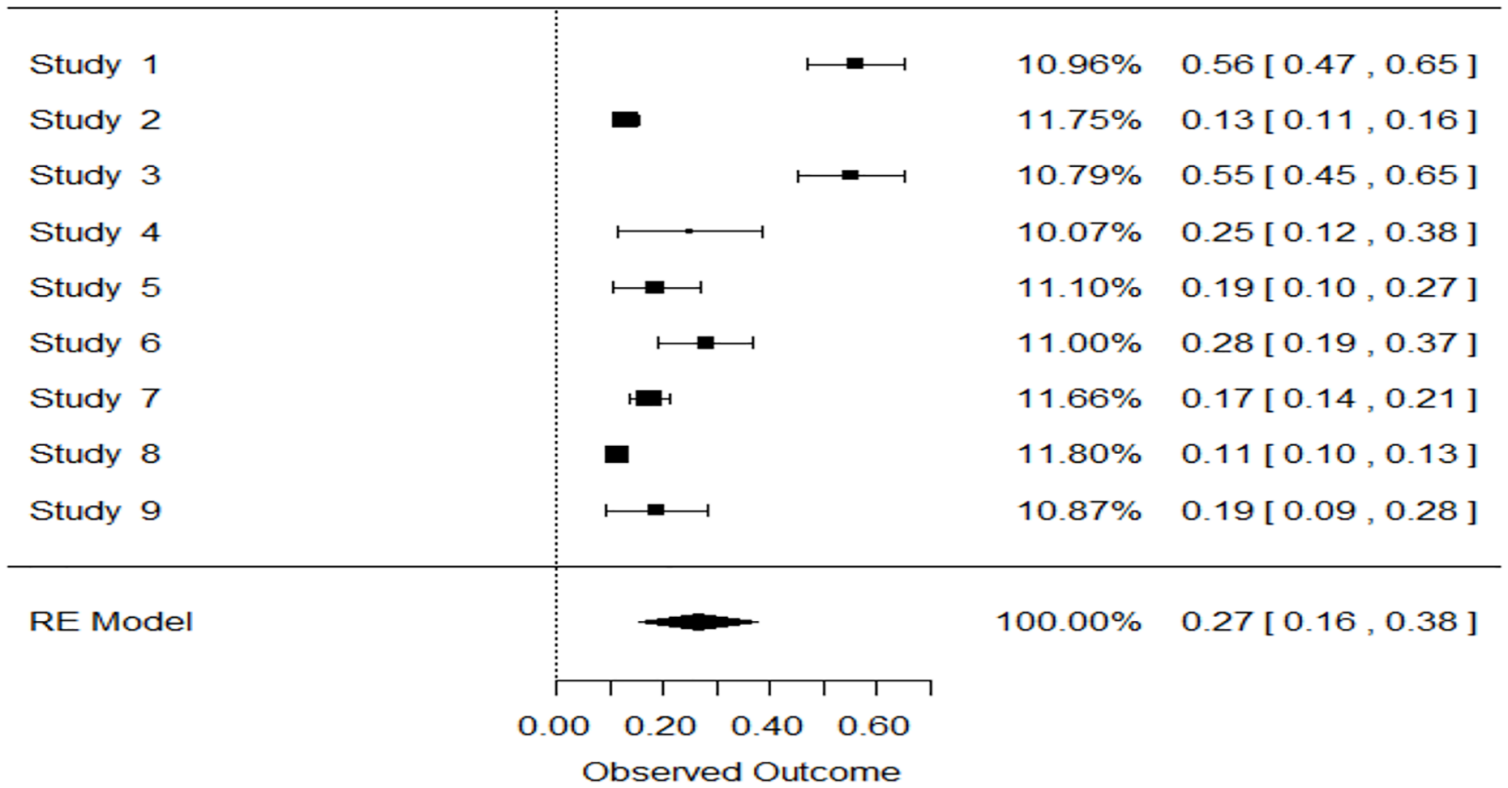
Forest Plot: polled prevalence of overlap among COPD patients in population based studies. Study 1 = Shirtcliffe et al [10], study 2 = Menezes et al [11], study 3 = Marsh et al [12], study 4 = Johannessen et al [16], study 5 = Danielsson et al [17], study 6 = Methvin et al [18], study 7 = Miravitlles et al [19], study 8 = Y. Zhou+CESCOPD et al [20], study 9 = Jyrki-Tapani al [21].

**Fig 3:**
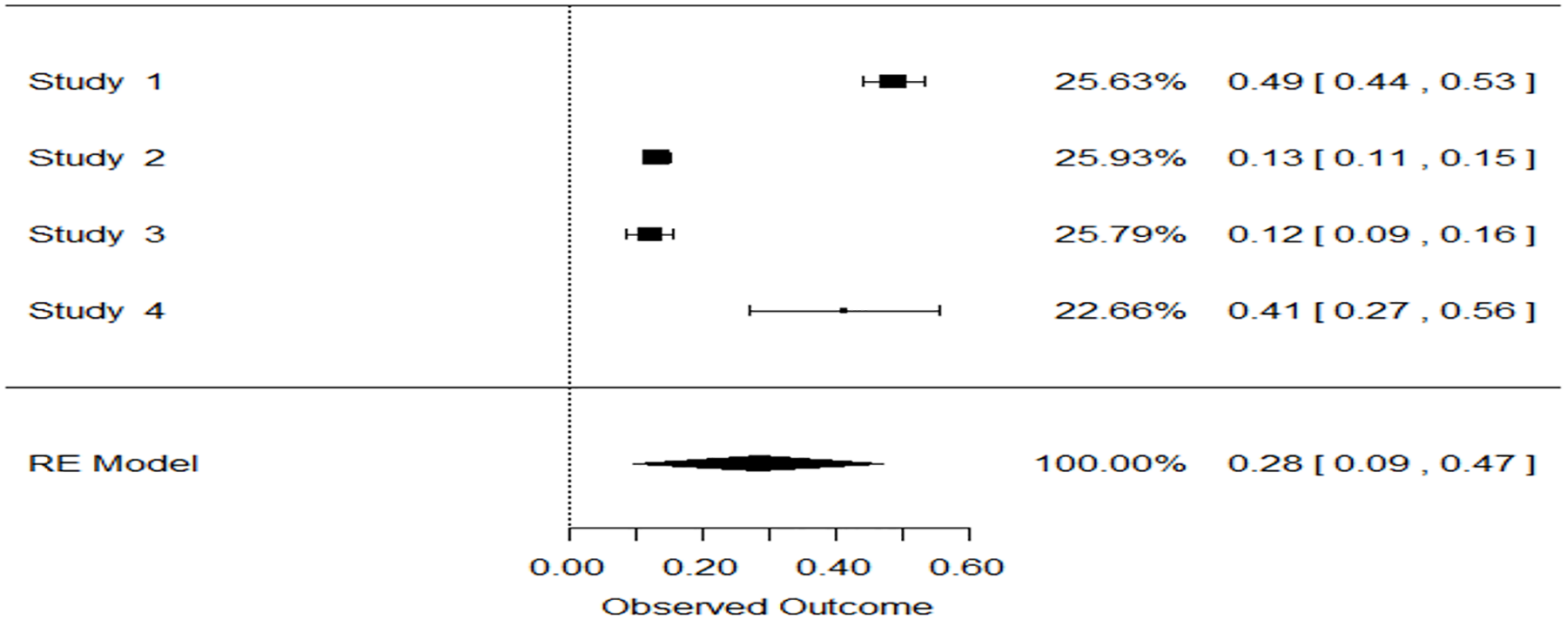
Forest Plot: polled prevalence of overlap among COPD patients in hospital based studies. Study 1 = Kauppi et al [13], study 2 = Hardin et al [14], study 3 = Alonso JL et al [15], study 4 = Fabbri et al [22].

One of the objectives of this study was to estimate prevalence of this overlap phenotype in COPD patients. Only 13 studies used post-BD values for COPD diagnosis. Data from these 13 studies were pooled using a random effects model, Figs 2 and 3. Pooled prevalence of overlap was 27% (95% CI: 0.16–0.38, p<0.0001) and 28% (95% CI: 0.09–0.47, p = 0.0032) in population and hospital based studies respectively.

### Age and Gender

The age of COPD and ACOS patients was reported in seven studies [11,13,14,19, 22–24]. All studies reported that ACOS subjects were younger than patients with only COPD, and this was statistically significant in five studies. Only two studies reported that ACOS patients were significantly older than patients with asthma alone with a mean (SD) age (in years) of 60.4 (11.3) and 61 (7) Vs. 54.9 (10.9) and 53 (13) (p<0.001)[11,13].

Five studies reported the gender of ACOS and COPD patients [11,13,14,19,22]. Our subgroup analysis showed that the difference in prevalence of ACOS among COPD patients for males versus females was-0.085 (95% CI: -0.178–0.008, p = 0.073).

### Healthcare Utilization and Cost

Five studies reported that patients with ACOS had more frequent exacerbations, hospitalizations, and emergency department visits in comparison to patients with COPD alone. In a study evaluating the PLATINO population, Menezes et al.[11] reported that ACOS was associated with higher risks for exacerbations (Prevalence Ratio (PR): 2.11; 95%CI 1.08–4.12), hospitalizations (PR 4.11; 95%CI 1.45–11.67), worse general health status (GHS) (PR 1.47; 95%CI 1.18–1.85), compared to those with COPD alone. Moreover, Hardin et al.[14]also reported that ACOS patients were more likely to have had a severe COPD exacerbation in the past year, and were more likely to experience frequent exacerbations (OR 3.55, CI: 2.19–5.75, p < 0.0001). Rhee et al.[23] reported that the percentages of emergency department visits, hospital admissions, and intensive care unit admissions were significantly higher among the ACOS patients compared to the COPD alone group (14.6%, 30.5%, and 0.5%, versus 5.0%, 14.1%, and 0.2%, p < 0.001). Similar significant findings were also observed in other studies [18,23–25]. In contrast, Alonso et al.[15] observed no significant differences between ACOS and other COPD phenotypes in terms of exacerbations, ED visits, or hospital admissions. However, the later study divided COPD patients into three different phenotypes using predefined and modified classification criteria which could explain the contrary findings.

Three studies compared the health care cost of ACOS and COPD subjects. The studies by Rhee et al.[23] and Shaya et al.[26] found that the total cost of all medical utilization of ACOS patients was 60% higher compared to COPD alone patients (p < 0.001). More importantly, they also reported that ACOS patients had higher cost in terms of inpatient and outpatient services (p < 0.001). ACOS was also significantly and independently associated with higher cost and utilization in a multivariate model (23). In addition, Blanchette et al.[24] reported that ACOS patients had higher adjusted respiratory-related total health care costs by 37.1% (P < 0.001). In comparison to patients with asthma alone, Shaya et al.[26] reported that ACOS patients cost five times (OR, 5.25; 95% CI, 4.59 to 6.02) more for total medical services. However, the study by Shaya et al. has certain limitations as it was confined to low-income populations, and female subjects.

### Health-Related Quality of Life (HRQoL)

Three studies reported the findings using Airway Questionnaire 20 (AQ20) and St. George’s Respiratory Questionnaire (SGRQ) (higher score means lower quality of life). In a logistic regression model, with the asthma group as reference, Kauppi et al.[13] reported that both the ACOS and the COPD group had low HRQoL (OR, 1.93; 95% CI: 1.16–3.2; and OR: 1.8; 95% CI: 1.0–3.2; respectively), but the quality of life of ACOS subjects was lower than COPD patients as reflected by the significantly higher AQ20 summary score (8.8 at the 1-year follow-up Vs. 7.4, p ≤ 0.05). The studies by Hardin et al.[14] and Miravitlles et al.[19] have observed similar findings and reported a significantly lower HRQoL in subjects with ACOS, as measured by SGRQ.

In relation to Body Mass Index (BMI), the previous three studies in addition to Menezes et al.[11] have reported that patients with overlap had higher mean BMI than patients with COPD only, and this was statistically significant in two studies[12,19].

### Medication Used

Rhee et al. (23) reported that inhaled corticosteroids (ICS), ICS plus long-acting beta-2 agonist (LABA), long-acting muscarinic antagonist (LAMA), leukotriene receptor antagonists (LTRA), oral corticosteroids (OCS), short-acting muscarinic antagonist (SAMA), short-acting beta-2 agonist (SABA), SAMA plus SABA, systemic beta agonists and theophylline were used more frequently in ACOS than in the COPD alone group (p < 0.001). Likewise, Alonso et al. [15] found that the use of the fixed dose combination of LABA and ICS was significantly higher in ACOS than in the pure COPD phenotype (p <0.05).

### Smoking status

The smoking status of participants was reported in 5 studies [11,13,14,19,22]. Four studies found no significant differences between the two groups in terms of being an active, ex, or never smoker. However, subjects with ACOS had fewer pack-years of smoking with three studies reporting a significantly (p<0.005) lower lifetime smoking history in individuals with ACOS [13,14,19].

### Pulmonary function, 6-minute walking distance, and imaging

Several studies compared lung function between COPD and ACOS subjects [11,13,14,19, 22], with pre and post-bronchodilator results included in only 4 studies. There was a wide variation in the lung function results reported. Menezes et al.[11] and Miravitlles et al.[19] observed that patients with ACOS had the lowest values for several lung function parameters, while Kauppi et al.[13] and Fabbri et al.[22] found better pulmonary function in ACOS patients. However, all the reported differences between the two groups were not statistically significant.

The 6mWD of ACOS subjects was reported in two studies [14,19] and compared the walk distance of COPD and ACOS subjects but did not find any significant difference between the two groups.

Only two studies reported CT findings of overlap patients. Hardin et al.[14] reported that subjects with asthma and ACOS demonstrated greater gas trapping on chest CT compared to patients with only COPD with no difference in CT measurements of emphysema or airway wall thickness. Fabbri et al. [22] observed higher diffusing capacity, PaO2, and lower emphysema score in asthmatics with fixed airflow obstruction. Both studies suggest that airway inflammation rather than parenchymal destruction is the major determinant in the decrease in pulmonary function in subjects with overlap.

## Discussion

The present systematic review provides the first quantitative summary of the world literature on ACOS. The aim of this review was to expand the understanding of the overlap between asthma and COPD by quantifying the prevalence and exploring the main characteristics of this overlap.

Subjects with ACOS represent a large proportion of COPD patients (~27%), and they form a distinct clinical phenotype with unique characteristics in comparison to patients with only COPD. These subjects are more likely to be younger, have less smoking history, with a high BMI, but exhibiting a similar degree of severity in terms of spirometry and the 6-minute walking distance. In addition, subjects with ACOS were more likely to have frequent and severe respiratory exacerbations than in those with either disease alone. However, the clinical distinction between the two obstructive diseases was not always clear.

The results of our review and meta analysis confirms and extends the complexity of this syndrome as it proves that patients with ACOS share the same demographic features, and exhibit similar patterns on pulmonary function test as patients with only COPD. More importantly, it indicates that the distinction between these two diseases cannot be based simply on spirometry testing, with or without bronchodilator response. As a result, both GINA and GOLD in a combined statement has recently published guidelines in order to assist clinicians to identify patients, who have chronic airflow limitation and distinguish subjects with asthma from COPD and ACOS, and to decide on initial treatment and/or need for referral[5]. In the absence of controlled trials recommendations can only be based on consensus but its clear that subjects with an asthma component should not be treated without an inhaled corticosteroids.

ACOS appears to share many of the same disease risk factors as that of COPD. These risk factors include smoking, advancing age, and history of asthma. The study by de Marco et al.[25] found that the prevalence of ACOS significantly increased with age (p<0.001). Similarly, in a logistic regression model of ACOS, Fu et al. [27] found that age was significantly associated with this specific phenotype (p = 0.012).

Several studies reported that asthma is a significant risk factor for the future development of COPD [10,20,28,29–35], of which three studies investigated risks in non-smoking COPD patients and found that asthma was an independent risk factor [31–33].

Data on the association of asthma duration and activity with risk of COPD have been reported in many studies. Compared with those with a prior history of asthma, Gershon et al.[34] found that subjects with current asthma were more likely to have COPD (p<0.001). The study by Shahab et al. (29) and Bridevaux et al.[30] reported the more severe the COPD, the more likely people were to volunteer a diagnosis of asthma (p<0.02). Moreover, Shirtcliffe et al.[10] reported that childhood asthma was the strongest predictor of GOLD-defined COPD with a comparison of odds ratios showing that this was equivalent to adding about 20 years to lung age or about 60 pack years of smoking.

The increase in exacerbation and hospitalization rates, and the poor quality of life could be explained by the presence of more co morbidities in ACOS patients. Many studies have found ACOS patients were more likely to have more comorbidities in comparison to patients with only COPD. The studies by Miravitlles et al. [19] and Blanchette et al.[24] reported a significantly higher mean Charlson Comorbidity Index (CCI) (p<0.001). Similarly, increased comorbidity was more frequently seen in the group with ACOS, as reported by Rhee et al.[23]. In contrast, Kauppi et al.[13] and Andersén et al.[36] did not report more comorbidity in overlap patients, despite having lower quality of life and higher hospitalization rate than patients with only COPD.

Previous studies have shown that individuals with concomitant diagnosis of COPD and asthma incur higher health care costs and utilize more health care services and postulated that increased morbidity may have contributed to the greater utilization of the medical services in overlap patients [23,24,26].

Considering their clinical characteristics, finding an increased mortality in patients with ACOS may not be surprising. However, we found contradictory results with regard to mortality in ACOS. Diaz-Guzman et al.[37] reported that patients with ACOS had a higher risk of death during follow-up (adjusted Hazard Ratio [HR] 1.45, 95% CI 1.06, 1.98). Meyer et al.[38] found adults dying with COPD were more likely than those dying without COPD to have a history of asthma (OR, 5.0; 95% CI, 3.2 to 7.8). Furthermore, Panizza et al.[39] reported chronic airflow obstruction in asthmatics is associated with increased risk of mortality. On the other hand, several studies have found that a concomitant diagnosis of asthma had a protective mortality effect among patients with COPD. Lundbäck et al.[40] reported longitudinal study of patients with COPD, found the mortality among subjects with concomitant asthma was significantly lower over 20 years of follow-up. The study by Mapel et al.[41] used longitudinally collected data to examine the relationship between two different pharmacological regimens and survival in COPD patients. They reported that a history of asthma was associated with reduced mortality. In addition, Soriano et al.[42] described greater survival rates in the subgroups of COPD patients with concomitant asthma. However, the latter study has certain limitations as it had short follow-up period (3 years).

Alonso et al.[15] and Rhee et al.[23] were the first studies to compare medication patterns between COPD and ACOS subjects. They reported that ICS+LABA and LTRA were used much more frequently in the ACOS subjects. The prescription pattern observed in the overlap syndrome was not surprising for two reasons. Firstly especially in primary care the differentiation between COPD and ACOS will not be easy and many patients with a suggestion of asthma in the past will have a tendency to have asthma like therapy. Secondly, patients with overlap syndrome have more severe disease as evident by greater health care utilization, which would cause physicians to incrementally increase the intensity of treatment.

At present, there are no randomized clinical trial data to help guide therapeutic interventions in ACOS, and this can be attributed to the fact that concomitant diagnosis of asthma and COPD is an exclusionary criteria in most if not all clinical trials. However, several studies have proposed management suggestions. Martinez et al.[43] has recommended treatment with a combination of ICS/LABA, with or without a long-acting anticholinergic agent. Similar suggestion was also proposed by Miravitlles et al.[44]. They reported that the overlap COPD-asthma phenotype is associated with enhanced response to inhaled corticosteroids due to the predominance of eosinophilic bronchial inflammation. Therefore, they suggest the prescription of inhaled corticosteroids together with long-acting bronchodilators irrespective of the severity of the airflow obstruction. Moreover, the GINA/GOLD joint document [5] recommends that if the syndromic assessment suggests asthma or ACOS, or there is significant uncertainty about the diagnosis of COPD, it is prudent to start treatment as for asthma until further investigation has been performed to confirm or refute this initial assessment, and the treatment regimen should be a combination of ICS and LABA. Surprisingly, only one study aimed at establishing the efficacy of ICS treatment in the overlap phenotype. The findings by Lim et al.[45] contradicts the current recommendations. They observed no beneficial effect of ICS on the annual rate of decline in FEV1, incidence rates of severe exacerbation, or overall mortality in ACOS patients. There are, however, some limitations to the previous study as it was a retrospective observational study with small sample size.

In another treatment study, Magnussen et al.[46] conducted a randomized, double-blind, placebo-controlled parallel group study with tiotropium for 12 weeks in patients with ACOS and significant differences were observed. At 12th week, tiotropium improved the primary endpoint FEV1 area under the curve (AUC) from 0 to 6 h (difference = 186 ± 24 ml, p<0.001) and for morning pre-dose FEV1 (difference = 987 ± 23 ml, p<0.001). Moreover, the mean weekly number of daily puffs of prn salbutamol was reduced by 0.50 ± 0.12 puffs/day in the tiotropium group (p<0.05). Given the now clear evidence for the efficacy of tiotropium in both asthma and COPD these results are not surprising.

### Strengths and limitations

There are certain limitations in our study. Firstly, although clinicians evaluated disease status in the majority of studies, objective measures and the absence of standardized diagnostic criteria for asthma diagnosis cast some doubt on the validity of the diagnosis. Secondly, we included studies that used different diagnostic criteria for ACOS. However, we believe it is defendable since there are no universally recognized criteria for diagnosing this syndrome. The strength of this study is that we have comprehensively collated the currently available literature on this rapidly emerging issue of major importance in the management of airways diseases.

### Conclusion

In summary, the findings of our review demonstrates the significant clinical impact and economic burden posed by ACOS, and emphasizes the need for clear diagnostic criteria and prospective validation with in parallel studies to define the optimal and cost effective management strategies for subjects with ACOS.

## Supporting Information

S1 PRISMA Checklist Research Checklist (PRISMA 2009 Checklist). (PDF)

S1 Fig Search process. (PDF)

S2 Fig The Modified Newcastle Ottawa scale for Cohort Studies. (PDF)

S3 Fig The Modified Newcastle Ottawa scale for Cross Sectional studies. (PDF)

S1 Table The quality assessment scores of selected studies. (DOCX)
