# Supplementary material for: Asthma and COPD Overlap Syndrome (ACOS): A Systematic Review and Meta Analysis
Source: PLoS One. 2015 Sep 3;10(9):e0136065. doi: 10.1371/journal.pone.0136065 (PMC4559416; doi:10.1371/journal.pone.0136065)
Supplement: S1 Fig — (PDF) [file pone.0136065.s002.pdf]

Search number	Search Terms*
**COPD**	
#1	"pulmonary disease, chronic obstructive"[MeSH Terms] OR "chronic obstructive pulmonary disease"[Text Word] OR "copd"[Text Word] OR "pulmonary emphysema"[MeSH Terms] OR "emphysema"[Text Word] OR "bronchitis, chronic"[MeSH Terms] OR "chronic bronchitis"[Text Word]
**Asthma**	
#2	"asthma"[MeSH Terms] OR "asthma"[Text Word]
**ACOS**	
#3	asthma-copd overlap syndrome[Text Word] OR overlap of asthma and copd[Text Word] OR asthma and chronic obstructive pulmonary disease overlap syndrome[Text Word] OR ACOS[Text Word] OR Asthma-Chronic obstructive pulmonary disease overlap syndrome[Text Word] OR overlap of asthma and chronic obstructive pulmonary disease[Text Word]
#4	(#1 OR #2 OR #3)
**Epidemiology Terms**	
#5	"epidemiology"[MeSH Terms] OR "incidence"[MeSH Terms] OR "prevalence"[MeSH Terms] OR ("hospital mortality"[MeSH Terms] NOT "hospital mortality/ethnology"[MeSH Terms]) OR "morbidity"[MeSH Terms]
#6	(#4 AND # 5)
#7	"pulmonary disease, chronic obstructive/epidemiology"[MeSH Major Topic] OR "pulmonary disease, chronic obstructive/mortality"[MeSH Major Topic] OR "pulmonary emphysema/epidemiology"[MeSH Major Topic]) OR "pulmonary emphysema/mortality"[MeSH Major Topic] OR "bronchitis, chronic/epidemiology"[MeSH Major Topic] OR "bronchitis, chronic/mortality"[MeSH Major Topic]) OR ("asthma/epidemiology"[MeSH Major Topic] OR "asthma/mortality"[MeSH Major Topic]
#8	(#6 OR #7)
**Exclusionary Terms**	
#9	"comment"[Publication Type] OR "review"[Publication Type] OR "editorial"[Publication Type] OR "letter"[Publication Type]) OR "clinical trial"[Publication Type]
**Total**	
#10	(#8 NOT #9)

Notes: *Search limits: English language, full text, and publication date from 1 January 1965 to February 01/2015.
