# Supplementary material for: Asthma and COPD Overlap Syndrome (ACOS): A Systematic Review and Meta Analysis
Source: PLoS One. 2015 Sep 3;10(9):e0136065. doi: 10.1371/journal.pone.0136065 (PMC4559416; doi:10.1371/journal.pone.0136065)
Supplement: S2 Fig — (PDF) [file pone.0136065.s003.pdf]

The modified Newcastle Ottawa scale for Cohort studies

Selection				Comparability	Outcome		
**Representativeness of the exposed cohort**	**Selection of the non exposed cohort**	**Ascertainment of exposure**	**Demonstration that outcome of interest was not present at start of study (for side effects)**	**Comparability of cohorts on the basis of the design or analysis**	**Assessment of outcome**	**Was follow-up long enough for outcomes to occur**	**Adequacy of follow up of cohorts**
a) Truly representative of the average ACOS in the community * <br>b) Somewhat representative of the average ACOS in the community * <br>c) Selected group of ACOS (e.g. smokers, non-smokers) <br>d) No description of the derivation of the cohort	a) Drawn from the same community as the exposed cohort * <br>b) Drawn from a different source <br>c) No description of the derivation of the non exposed cohort	a) Secure record (e.g. clinical records) * <br>b) Structured interview * <br>c) Written self report <br>d) No description	a) Yes * <br>b) No	a) Most important factors of adjustments (age, gender, asthma history, smoking, initial FEV1) * <br>b) Any additional factors (level of education, socio-economic status ethnicity/race) *	a) Independent blind assessment * <br>b) Record linkage * <br>c) Self report <br>d) No description	a) Yes (10 years) * <br>b) No	a) Complete follow up - all subjects accounted for * <br>b) Subjects lost to follow up unlikely to introduce bias (lost to follow up < 10 %) <br>c) Follow up rate < 10 % and no description of those lost <br>d) No statement
