# Supplementary material for: Asthma and COPD Overlap Syndrome (ACOS): A Systematic Review and Meta Analysis
Source: PLoS One. 2015 Sep 3;10(9):e0136065. doi: 10.1371/journal.pone.0136065 (PMC4559416; doi:10.1371/journal.pone.0136065)
Supplement: S3 Fig — (PDF) [file pone.0136065.s004.pdf]

The modified Newcastle Ottawa scale for cross sectional studies

Selection				Comparability	Outcome	
**Representativeness of the sample**	**Sample size**	**Ascertainment of exposure**	**Non-respondents**	**The subjects in different outcome groups are comparable, based on the study design or analysis. Confounding factors are controlled.**	**Assessment of outcome**	**Was follow-up long enough for outcomes to occur**
a) Truly representative of the average ACOS in the community * <br>b) Somewhat representative of the average ACOS in the community * <br>c) Selected group of ACOS (e.g. smokers, non-smokers) <br>d) No description of the derivation of the cohort	a) Justified and satisfactory. * <br>b) Not justified.	a) Validated measurement tool. * <br>b) Non-validated measurement tool, but the tool is available or described. * <br>c) No description of the measurement tool.	a) Comparability between respondents and non-respondents characteristics is established, and the response rate is satisfactory. * <br>b) The response rate is unsatisfactory, or the comparability between respondents and non-respondents is unsatisfactory. <br>c) No description of the response rate or the characteristics of the responders and the non-responders.	a) The study controls for the most important factor (Childhood asthma). * <br>b) The study control for any additional factor (age, gender, ethnicity). *	a) Independent blind assessment * <br>b) Record linkage * <br>c) Self report <br>d) No description	a) The statistical test used to analyze the data is clearly described and appropriate, and the measurement of the association is presented, including confidence intervals and the probability level (p value). * <br>b) The statistical test is not appropriate, not described or incomplete.
