# Supplementary material for: Asthma and COPD Overlap Syndrome (ACOS): A Systematic Review and Meta Analysis
Source: PLoS One. 2015 Sep 3;10(9):e0136065. doi: 10.1371/journal.pone.0136065 (PMC4559416; doi:10.1371/journal.pone.0136065)
Supplement: S1 Table — (DOCX) [file pone.0136065.s005.docx]

**S1 Table. The quality assessment scores of selected studies**

| **STUDY** | **SELECTION** | **COMPAIRABILITY** | **OUTCOME** | **TOTAL** |
| --- | --- | --- | --- | --- |
| **Shirtcliffe et all (2010)** | **3** | **2** | **2** | **7** |
| **Menezes et al (2013)** | **3** | **1** | **2** | **6** |
| **Marsh et al (2008)** | **3** | **0** | **2** | **5** |
| **Kauppi et al (2011)** | **3** | **1** | **3** | **7** |
| **Hardin et al (2011)** | **2** | **1** | **2** | **5** |
| **Alonso et al (2013)** | **3** | **0** | **2** | **5** |
| **Johannessen et al (2005)** | **4** | **1** | **1** | **6** |
| **Danielsson et al (2010)** | **4** | **1** | **2** | **7** |
| **Methvin et al (2008)** | **2** | **1** | **2** | **5** |
| **Miravitlles et al (2013)** | **3** | **0** | **2** | **5** |
| **Zhou et al (2009)** | **2** | **1** | **2** | **5** |
| **Kotaniem et al (2005)** | **3** | **1** | **2** | **6** |
| **Fabbri et al (2008)** | **3** | **1** | **2** | **6** |
| **Rhee et al (2013)** | **3** | **1** | **2** | **6** |
| **Blanchette et al (2008)** | **3** | **1** | **2** | **6** |
| **de marco et al (2013)** | **3** | **1** | **1** | **5** |
| **Shaya et al (2008)** | **3** | **1** | **2** | **6** |

Based on the Newcastle-Ottawa scale (NOS)[9].
